# A Software Architecture to Mimic a Ventricular Tachycardia in Intact Murine Hearts by Means of an All-Optical Platform

**DOI:** 10.3390/mps2010007

**Published:** 2019-01-08

**Authors:** Francesco Giardini, Valentina Biasci, Marina Scardigli, Francesco S. Pavone, Gil Bub, Leonardo Sacconi

**Affiliations:** 1 European Laboratory for Non-Linear Spectroscopy, 50019 Sesto Fiorentino, Italy; giardini@lens.unifi.it (F.G.); biasci@lens.unifi.it (V.B.); scardigli@lens.unifi.it (M.S.); pavone@lens.unifi.it (F.S.P.); 2 Division of Physiology, Department of Experimental and Clinical Medicine, University of Florence, 50134 Florence, Italy; 3 National Institute of Optics, National Research Council, 50125 Florence, Italy; 4 Department of Physiology, McGill University, Montreal, QC H3A 0G4, Canada; gilbub@gmail.com

**Keywords:** optical mapping, voltage imaging, voltage sensitive dye, optical manipulation, optogenetics, channelrhodopsin-2, cardiac electrophysiology, LabVIEW, closed-loop, real-time analysis

## Abstract

Optogenetics is an emerging method that uses light to manipulate electrical activity in excitable cells exploiting the interaction between light and light-sensitive depolarizing ion channels, such as channelrhodopsin-2 (ChR2). Initially used in the neuroscience, it has been adopted in cardiac research where the expression of ChR2 in cardiac preparations allows optical pacing, resynchronization and defibrillation. Recently, optogenetics has been leveraged to manipulate cardiac electrical activity in the intact heart in real-time. This new approach was applied to simulate a re-entrant circuit across the ventricle. In this technical note, we describe the development and the implementation of a new software package for real-time optogenetic intervention. The package consists of a single LabVIEW program that simultaneously captures images at very high frame rates and delivers precisely timed optogenetic stimuli based on the content of the images. The software implementation guarantees closed-loop optical manipulation at high temporal resolution by processing the raw data in workstation memory. We demonstrate that this strategy allows the simulation of a ventricular tachycardia with high stability and with a negligible loss of data with a temporal resolution of up to 1 ms.

## 1. Introduction

**Figure 1:**
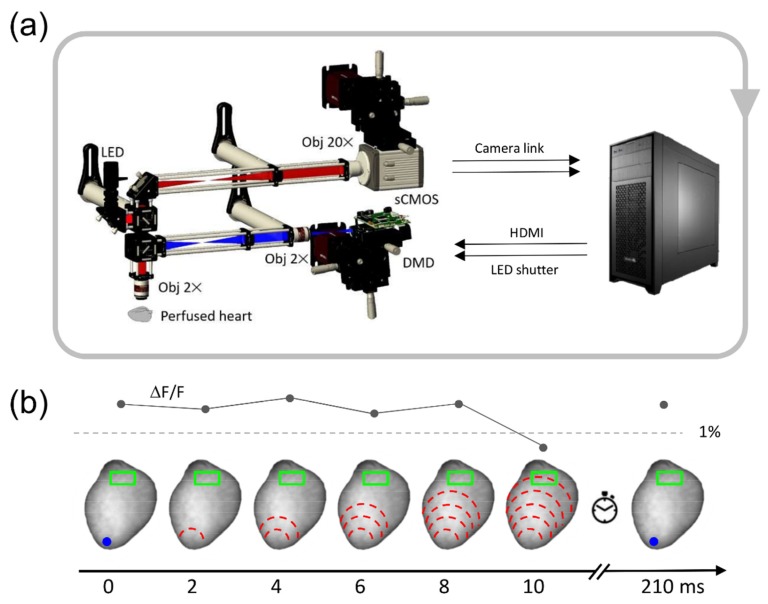
Real-time architecture to mimic ventricular tachycardia. (**a**) Scheme of the wide-field fluorescence mesoscope in tandem with the workstation for real-time analysis and manipulation. Figure modified from [21]. LED, light-emitting diode; sCMOS, scientific complementary metal-oxide semiconductor; DMD, digital micro-mirror device; HDMI, high-definition multimedia interface. (**b**) At the top panel, the trace shows the corresponding fluorescent signal value in the detection region of interest (ROI; green rectangle). When the signal overcomes a set threshold (1%) the system closes the loop reinjecting a light stimulus. To the bottom, channelrhodopsin-2 (ChR2) stimulation at ventricle apex (blue spot) is applied to optically activate the heart. The wavefront propagation (dashed red lines) propagates from the apex to the base of the heart. When the propagating wave invades the detecting ROI (green rectangle) the system reinjects the light stimulus at the apex of the heart with a pre-defined delay (clock). The simulated ventricular tachycardia can be initiated by a single external optical or electrical stimulus or a spontaneously generated sinus beat.

Optogenetics is an emerging technique that involves the use of light to control the electrical activity of excitable cells. Expression of light-sensitive depolarizing ion channels such as ChR2 in excitable cells enables a fast-excitatory response to illumination [1,2,3]. From applications in neuroscience, optogenetics has been widely extended to cardiac research [4,5,6,7,8,9,10,11,12,13,14]. Several therapeutic approaches based on optogenetics have recently been reported: Nussinovitch and Gepstein [15] described the first use of optogenetics for cardiac resynchronization while other groups [16,17,18,19,20] have demonstrated optical defibrillation of arrhythmic hearts. In this context, Crocini and colleagues [19] developed an optical platform that can generate novel stimulation strategies with the aim of developing new cardiac defibrillation strategies: a wide-field mesoscope to map the action potential propagation across perfused mouse hearts was implemented with an ultrafast scanning laser system to manipulate cardiac electrical activity with arbitrarily chosen channelrhodopsin-2 (ChR2) stimulation patterns. This optical toolkit enabled full optical control of cardiac conduction at sub-ms temporal resolution in whole isolated mouse hearts. However, an important feature was missing: the capability to interact with the cardiac tissue based on the ongoing electrical activity. We recently addressed this issue by using a closed-loop approach where the stimulation was directly triggered by cardiac dynamics in real-time in intact hearts [21]. Our approach uses a computer-controlled digital micro-mirror device (DMD) to control cardiac excitation waves in real time with pre-defined stimulation pattern (Figure 1A). The system was successfully used to manipulate intraventricular propagation, atrioventricular delay, and to mimic pathologies by imposing a re-entrant circuit in a healthy heart. In the latter example, a light pulse delivered to the apex of a ChR2 expressing heart induced a wave that propagated to the heart base, and once an action potential was optically detected in a selected region of interest, a new trigger was generated in the apex with a certain delay (Figure 1B). This experiment was the first demonstration of an all-optical system that can generate a user defined re-entrant circuit in an intact heart preparation. Here, we describe an improved version of the software that can optically mimic a ventricular tachycardia with high stability, negligible data loss, and with unprecedented temporal resolution.

## 2. Materials and Methods

**Optical platform.** As described in [21], the whole mouse heart was illuminated in wide-field configuration using a 2× objective and a LED (light-emitting diode) operating at a wavelength centered at 625 nm, followed by a band-pass filter at 640/40 nm. A dichroic beam splitter followed by a band-pass filter at 775/140 nm were used for collecting the emitted fluorescence from a voltage-sensitive dye (VSD) used for staining the heart. A 20× objective was used to focus the fluorescence in a central portion (128 × 128) px of the sensor of a scientific complementary metal-oxide semiconductor camera (sCMOS camera), corresponding to a field of view of about (10 × 10) mm in the sample plane. The camera is set to run in free-run mode, with frame rate determined by exposure time. A Texas Instruments Lightcrafter 4500 projector (Dallas, TX, USA), containing a DMD, was used to manipulate electrical activity of the heart by projecting user-defined light patterns onto the heart. All microscope components were fixed onto a custom vertical honeycomb steel breadboard. The workstation is a Dell running a 64 bits version of Windows 7 Professional, 32 GB RAM and an Intel Xeon processor E5-1630 v3 at 3.70 GHz. For all details, see [21].

**Sample preparation.** All animal handling and procedures were performed in accordance with the guidelines from the Directive 2010/63/EU of the European Parliament on the protection of animals used for scientific purposes. The experimental protocol was approved by the Italian Ministry of Health (protocol number 647/2015-PR). As described in [21], transgenic mice were heparinized (5000 units/mL) and anesthetized by inhaled isoflurane (5%). The excised heart was immediately bathed in Krebs–Henseleit (KH) solution and cannulated through the aorta. Contraction was inhibited with blebbistatin (5 μM) in the solution. The cannulated heart was retrogradely perfused (Langendorff perfusion) with the KH solution and then transferred to the recording chamber at a constant flow of 2–5 mL/min at 22 °C (room temperature). After few minutes, 1 mL of perfusion solution containing the voltage sensitive dye (di-4-ANBDQPQ, 50 µg/mL) [22] was bolus injected into the aorta.

## 3. Software Architecture

### 3.1. General Architecture and Funcionality

We developed a single custom LabVIEW software program (LabVIEW 2015, Version 15.0 64-bit, National Instruments, Austin, TX, USA) to implement the optical platform described in Scardigli et al. [21]. The goal of this solution is to simultaneously manage the sCMOS camera system (OrcaFlash4.0, Hamamatsu, Shizuoka, Japan) for fast imaging, the real-time image analysis and the DMD-based optical stimulator (DLPLCR4500EVM, Texas Instruments, Dallas, TX, USA). In order to achieve fast feedback control, camera management has been integrated in the main LabVIEW program using the Hamamatsu Video Capture Library (HVCL, Version 4.2, 2017; Hamamatsu City, Shizuoka, Japan), so the raw data from the camera is directly fast stacked and instantly analyzed in memory, without incurring time expensive external backups. The program allows two operating modalities: free-run mode (i.e., optical mapping without active control) and closed-loop mode. Both modalities exploit the 16-bit performance of our sCMOS sensor and it is possible to switch between these modes at any point during the experiment. The closed-loop modality was developed to mimic a re-entrant ventricular tachycardia by performing the real-time optical mapping and stimulation protocol described above.

**Figure 2:**
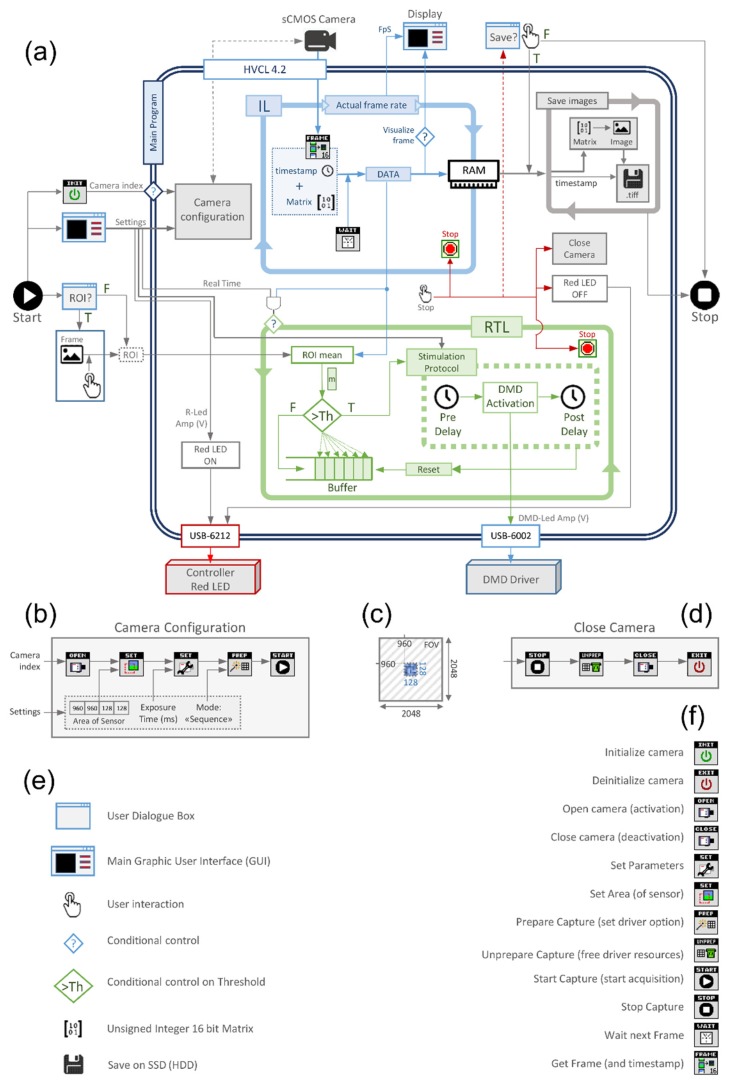
Conceptual Scheme of the LabVIEW Program Architecture. (**a**) Scheme of the LabVIEW program that manages the whole optical-mapping setup. The main program is principally composed by two loops: the IL (imaging loop) in blue and the RTL (real-time loop) in green. LED, light-emitting diode; sCMOS, scientific complementary metal-oxide semiconductor; DMD, digital micro-mirror device; HVCL, Hamamatsu video capture library; ROI, region of interest; FpS, frame per second; RAM, random access memory; R-Led, red LED; Amp (V), amplitude (Volts); USB, universal serial bus; F, false; T, true; Th, threshold; TIFF, tagged image file format. (**b**) Camera configuration. This block contains the HVCL functions to prepare the camera. (**c**) Representation of the portion of the camera sensor used. FOV, field of view. (**d**) Close camera. This block contains the operations to correctly stop and close the connection with the camera. (**e**) Legend of the symbol used in panel (**a**). SSD, solid state drive; HDD, hard disk drive. (**f**) List of the Hamamatsu Video Capture Library (HVCL) functions used.

The software is based on two parallel asynchronous loops: the imaging loop (IL in Figure 2A), and the real-time analysis loop (RTL in Figure 2A). The first manages the camera acquisition, stacks the camera data in RAM and controls the effective system frame rate. The second loop manages the stimulation protocol based on the analysis of the current camera data. In this configuration, system stability is guaranteed because the RTL is faster than IL. We decide to use two independent loops to allow image acquisition during all phases of the optically-induced re-entrant circuit. The analysis is based on a region of interest (ROI) selection in the camera frame; the ROI is set by the user before the real-time operation. The graphical user interface (GUI) allows also to set the following parameters: the exposure time of the camera, excitation LED (red LED) current, action potential depolarization threshold (percentage of ΔF/F, where F represents the VSD fluorescence signal) within the selected ROI, DMD LED (blue LED) current, stimulation pulse length and delay. The system first performs some preliminary operations to prepare the sCMOS camera and turn on the red LED before starting the imaging loop. When the closed-loop modality is active, the real time analysis is performed with every new image acquisition. Finally, the system manages the operations for correctly closing the camera connection and saving all the frames stacked in memory. A detailed description of these operating modalities is found in the following paragraphs. The LabVIEW software is available at: https://cuoricino.fisica.unifi.it/share.cgi?ssid=0hGs1Tb.

### 3.2. Preliminary Operations

When the program starts, the user can select a ROI in a pre-existing image by drawing a rectangle on a heart region (for example, at the base of the heart). Contemporaneously, the program initializes the camera executing INITIALIZE function from HVCL. Inside the main program, the first operation is to turn on the red LED (Figure 2A): the LED voltage is read by the GUI and sent by a multi-function I/O device (USB-6212, National Instruments, Austin, TX, USA) to the LED driver. Afterwards, based on the camera index, the camera is opened (executing the OPENCAMERA function from HVCL) and only the central 128 × 128 px area of the 2048 × 2048 px sensor is set (Figure 2B,C) and employed (executing the SETAREA function from HVCL) allowing fast imaging [21]. Furthermore, a generic SETPARAMETER function is used to set the sensor exposure time, while the PREPARECAPTURE function sets the acquisition in “sequence” modality (free run acquisition). Finally, the STARTCAPTURE function begins capturing of image data from the camera and IL is started.

### 3.3. Imaging Loop

As mentioned above, the IL manages the camera acquisition, stacks the data in RAM and controls the real frame rate of the system. Here, the GETFRAME function (Figure 2A) reads from the camera the current frame (managed as 128 × 128 unsigned 16-bit matrix) and the timestamp (a camera time reference for the current frame). Then WAITNEXTFRAME function waits until GETFRAME gives the new frame: when a new frame and timestamp are ready, these are written in a local variable and stacked in RAM. After this, the WAITNEXTFRAME function waits for the next frame from the camera and the cycle repeats itself. The loop also allows the visualization of the current frame in the GUI when the “visualize frame” option is set. This option is very CPU expensive and it is generally disabled during fast imaging and real-time analysis. The IL continually checks the actual frame rate using the workstation timestamp that is compared with the previous one at every cycle using a dedicated shift register.

### 3.4. Real-Time Analysis Loop

Real-time analysis loop is the core of the main program, and it is executed by setting the RT option in the GUI. When the first frame is written in local memory, the program calculates the average (m) of the fluorescence signal within the user-defined ROI (see Figure 2). At the first cycle, the shift register buffer is empty, so m is written in the buffer. By the second cycle the loop starts its comparative procedure to calculate the fluorescence ratio (ΔF/F). The current m value is compared with the m-values stored in the buffer: m is subtracted from the buffer vector and the result is divided by the buffer itself producing a relative variation vector. If one value of this resulting vector is bigger than the user-defined threshold (Th) a depolarization event is detected in the ROI area and a stimulation protocol is triggered. Stable operation is guaranteed when RTL is faster than IL (timed by the exposure time), where the comparison procedure is performed with each frame.

### 3.5. Stimulation Protocol

When a depolarization event is detected, the platform optically stimulates the sample with the blue LED, using the DMD-based Lightcrafter displaying a pre-defined pattern [21]. The stimulation protocol is composed of three sequential steps: a pre-activation delay, LED activation and a post-activation delay. The pre-activation delay is the time between the detection event and the optogenetic stimulation. Then, the platform illuminates the heart with a pulse of light: the driving voltage of blue LED is read by the GUI and sent to a multi-function I/O device (USB-6002, National Instruments, Austin, TX, USA) together with an enable digital command for the duration of the pulse width. Lastly, the system waits for a post-activation delay before restarting the ROI analysis; this post-activation delay guarantees that the system does not detect unwanted fluorescence variation related to optogenetic stimulation. Finally, the buffer is reset and the RTL looks for a new depolarization event.

### 3.6. Final Operations

At the end of the measurement, IL and RTL can be stopped using a dedicated button in the GUI. After loops are interrupted, a set of operations are necessary to correctly close the camera (Figure 2D): (i) the HVCL STOPCAPTURE function stops the acquisition of the camera; (ii) UNPREPARECAPTURE makes available all resources created with PREPARECAPTURE; (iii) CLOSECAMERA closes the camera; and (iv) finally, DEINITIALIZECAMERA function deactivates the camera and makes available all resources used by the video capture library. The system also turns off the red LED (by setting the LED current to zero) and the program queries the user to save all the acquired frames. After the prompt, a save image loop starts and every frame matrix stored in RAM is converted to a 16-bit 128 × 128 grayscale tiff image and saved to solid-state disk. The timestamp of each frame is used as its filename, and when all frames are saved the program ends.

## 4. Results and Discussion

**Figure 3:**
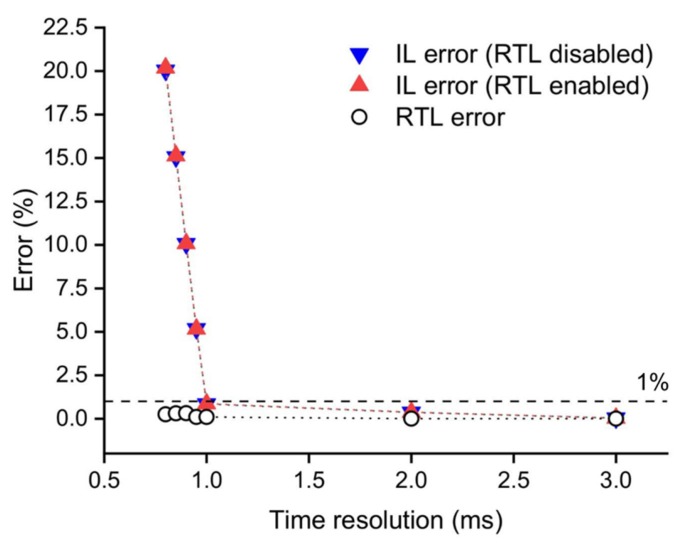
System performances at different time resolutions during 90 s of measurement. Imaging loop (IL) error rate calculated as the fraction of lost frames respect to total with real-time analysis loop (RTL) disabled (blue triangle) and enabled (red triangle). Real-time analysis loop error (black circle) calculated as the fraction of not analyzed frames respect to acquired frames.

Here, we evaluated the performance of our hardware and software architecture in terms of speed and stability during free-run and closed-loop imaging modalities. When reducing the frame integration time, the data transfer capability and workstation resources may not be sufficient to perform all required operations and a frame can be accidentally skipped during acquisition and/or RT analysis. A stress test was developed in order to estimate the relation between time resolution (exposure time) and the error rate of both IL and RTL. We have first measured IL performance calculating the fraction of lost frames during 90 s of recording applying different exposure times. The test was performed without and with the RT analysis (blue and red triangle respectively in Figure 3) to quantify the impact of the RTL execution during image acquisition. We found that with exposure times ≥1 ms the error rate is less than 1% for both configurations (RTL disabled and RTL enabled). Then, using a similar approach, we quantified the RTL performance measuring the fraction of the acquired frames that have not been analyzed during 90 s of RT protocol (black circles in Figure 3). We found an error <0.4% for each exposure time. Based on this observation, the data transfer capability represents the bottleneck of our system, and we can define a time resolution limit as 1 ms.

**Figure 4:**
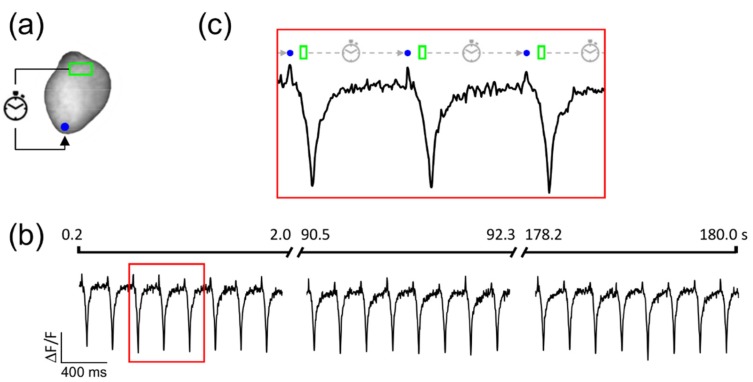
Stability of simulated ventricular tachycardia. (**a**) Fluorescent image of the heart. After detection of depolarization activity at the heart base (green ROI), the system stimulates ChR2 at ventricle apex (blue circle) using a delay of 200 ms. (**b**) Fluorescent signal (∆F/F) extracted from the green ROI shows a stable ventricular tachycardia during the entire time of the measurement (3 min). Note that the fluorescence of the di-4-ANBDQPQ voltage-sensitive dye (VSD) decreases during each membrane depolarization. (**c**) Zoom of the trace in panel (**b**): the green rectangle indicates the detection time of membrane depolarization, the dashed line indicates the delay period, and the blue circle indicates the time of ChR2 stimulation.

We tested the stability of the system over a three-minute recording window with a temporal resolution of 2 ms. The heart was optogenetically stimulated at the apex using a circular spot (1 mm in diameter) and a detection ROI (0.1 × 0.3 mm) was placed at the heart base (Figure 4a).

The used experimental parameters were as follows: illumination intensity 1 mW/mm^2^; optogenetic stimulation intensity 4 mW/mm^2^; pulse duration 5 ms; pre-activation delay 200 ms; post-activation delay 5 ms; detection threshold 1%. As fully described in Scardigli et al. [21], a threshold of 1% of ΔF/F was chosen based on a typical shot noise at rest within our ROI of 0.2–0.02% of ΔF/F and a VSD sensitivity of ~4%. Figure 4b shows the optical trace obtained. The stability of the system in different preparations was confirmed by successfully simulating ventricular tachycardia in five hearts using different recording durations (from 25 s to 3 min).

As described above, the system stores all images in RAM during imaging and processing and it saves the entire dataset at the end of the measurement. Although this approach results in a very high temporal resolution, it could limit the measuring time. For example, acquiring (128 × 128) px 16-bit images at 1 kHz uses 1.875 GB of RAM for every minute of recording.

The ability to stably mimic re-entrant circuits can be leveraged to generate biological models of different pathological conditions. Different delays and /or different stimulation patterns can be tested in order to fully characterize the stability of ventricular tachycardias in healthy or pathological substrates [23,24,25]. Optogenetics could be also employed to manipulate the conduction velocity and/or action potential duration using low light intensity illumination [7]. For example, selected areas of the heart could be statically illuminated with blue light inducing heterogeneities in conduction velocity and refractoriness during RT fixed delay stimulation, in order to mimic the effects of pathological substrates on re-entrant tachycardias.
